# Role of periostin in inflammatory bowel disease development and synergistic effects mediated by the CCL5–CCR5 axis

**DOI:** 10.3389/fimmu.2022.956691

**Published:** 2022-10-20

**Authors:** Saida Mukanova, Anton Borissenko, Alexey Kim, Aigerim Bolatbek, Ainur Abdrakhmanova, Luca Vangelista, Eva Sonnenberg-Riethmacher, Dieter Riethmacher

**Affiliations:** ^1^ Department of Biomedical Sciences, School of Medicine, Nazarbayev University, Astana, Kazakhstan; ^2^ School of Medicine, Nazarbayev University, Astana, Kazakhstan; ^3^ Department of Human Development and Health, School of Medicine, University of Southampton, Southampton, United Kingdom

**Keywords:** periostin (POSTN), CCL5-CCR5-signaling system, IBD – inflammatory bowel disease, inflammation, treatment option, chemically induced colitis

## Abstract

Inflammatory bowel disease (IBD), comprising mainly Crohn’s disease (CD) and ulcerative colitis (UC), is a chronic inflammatory disease of the gastrointestinal tract. In recent years, a wealth of data has been accumulated demonstrating the complex interplay of many different factors in the pathogenesis of IBD. Among these are factors impacting the epithelial barrier function, including vessel and extracellular matrix (ECM) formation, the gut microbiome (e.g., bacterial antigens), and, most importantly, the production of cytokines (pro- and anti-inflammatory) directly shaping the immune response. Patients failing to resolve the acute intestinal inflammation develop chronic inflammation. It has been shown that the expression of the matricellular protein periostin is enhanced during IBD and is one of the drivers of this disease. The C-C chemokine receptor 5 (CCR5) is engaged by the chemotactic mediators CCL3/MIP-1α, CCL4/MIP-1β, and CCL5/RANTES. CCR5 blockade has been reported to ameliorate inflammation in a murine IBD model. Thus, both periostin and CCR5 are involved in the development of IBD. In this study, we investigated the potential crosstalk between the two signaling systems and tested a highly potent CCL5 derivative acting as a CCR5 antagonist in a murine model of IBD. We observed that the absence of periostin influences the CCR5-expressing cell population of the gut. Our data further support the notion that targeted modulation of the periostin and CCR5 signaling systems bears therapeutic potential for IBD.

## Introduction

Inflammatory bowel disease (IBD) comes in two main forms, namely, Crohn’s disease (CD) and ulcerative colitis (UC), where CD is characterized by inflammatory lesions affecting any part of the gastrointestinal tract, while UC is typically confined to the colon (1). In the last global burden of disease (GBD) report from 2017, it was concluded that the prevalence of IBD had risen substantially during the last 20 years, and in 2017, there were about 7 million cases worldwide (2). This rise in the number of IBD cases is mainly due to an increase in newly industrialized countries, while the level in industrialized countries seems to have leveled on a high plateau (3). In most cases, IBD is a multifactorial disease influenced by genetic, immunological, and environmental factors as well as by the microbiome (4). More than 160 loci are known to confer susceptibility to CD and/or UC (4, 5). Interestingly, genes with the strongest associations are involved in the immune response to microorganisms, such as innate sensing of bacteria (NOD2), the inflammatory response to microbes (IL23R), and autophagy (ATG16L1) (3, 5). Rare monogenic disorders that affect intestinal immune and epithelial function can lead to early or very early onset IBD and severe phenotypes (6). Nevertheless, the attributable risk of heritability is relatively low, and twin studies demonstrate higher concordance for CD and only weak concordance for UC (7). The gut microbiota produce a range of small molecules from various metabolite classes with distinct biological effects, some of which modulate the gut immune homeostasis. Additionally, it is known that host-derived primary bile acids (BAs) are converted by resident microbes to secondary BAs and recent findings showed that BAs modulate the differentiation and function of T cells, including inflammatory TH_17_ cells and anti-inflammatory regulatory T (Treg) cells, which help to protect against extracellular pathogens and maintain host immune tolerance, respectively (8).

Matricellular proteins, as defined by Bornstein, can influence the microenvironment, the behavior of surrounding cells, and the homeostasis of tissues by binding to other matrix proteins as well as to cell surface receptors or to cytokines and proteases that interact with the cell surface (9). Periostin has recently emerged as a major matricellular protein player and driver in many inflammatory processes and also influenced the migration of cells (10–12). As a non-structural matricellular protein, periostin, initially called osteoblast-specific factor-2 (OSF-2), was originally cloned from the mouse osteoblastic cell line MC3T3-E1 (13). Periostin contains an N-terminal secretory signal peptide, followed by a cysteine-rich domain (EMI domain), four FAS1 domains, and a hydrophilic carboxy-terminal domain (CTD) that shows a remarkable amount of alternative splicing (12, 14). Recent studies have demonstrated that periostin plays a role in IBD; however, the precise mechanisms through which it influences the immune signaling pathways in IBD in patients and animal models are still not fully understood (15–18).

CCR5 is a seven-transmembrane, G protein-coupled receptor (GPCR) regulating the trafficking and effector functions of memory/effector T lymphocytes, macrophages, and immature dendritic cells (19). Many studies over the last two decades have demonstrated that CCR5 and its ligands play important roles in several biological processes spanning from HIV entry to cancer signaling and also including several inflammatory diseases (20). The first study demonstrating the involvement of CCR5 signaling in the dextran sodium sulfate (DSS)-induced murine colitis model reported that CCR5-deficient mice were protected from severe inflammation and concluded that CCR5 is critical for the promotion of intestinal Th1-type immune responses in mice during DSS-induced colitis (21). Another study reported that maraviroc (MVC), an orally active CCR5 antagonist, attenuated the development of intestinal inflammation in mice by selectively reducing the recruitment of CCR5-bearing leukocytes in the 2,4,6-trinitrobenzene sulfonic acid (TNBS) and DSS models (22). Additionally, it was recently shown that the CCL5:CCR5 axis seems to be involved in CD mucosal healing. When applying the CD-enriched *Debaryomyces hansenii* to DSS-treated mice, CCL5-neutralizing antibodies were able to rescue the wound repair defect supporting the conclusion that CCR5 was required for the detrimental effects of the fungus (23). Therefore, blockade of the CCL5:CCR5 axis seems to be a viable therapeutic avenue for IBD, and a previously described potent CCR5 antagonist (CCL5 5p12 5m) (24) may turn instrumental to this aim.

In this study, we analyzed whether there are interactions between the periostin and the CCL5:CCR5 systems in chemically induced colitis. In order to do this, we first monitored the level of CCR5-expressing cells in healthy as well as in inflamed tissues in periostin knockout (ko) and wild-type (wt) mice. In a second approach, we tested two CCR5 antagonists (MVC and CCL5 5p12 5m) in the chemically induced colitis context. Both the absence of periostin and applying CCR5 antagonists were able to reduce the number of CCR5-expressing cells and ameliorated the colitis phenotype. Thus, our results demonstrate that reducing the signaling in both systems holds therapeutic potential and that the absence of periostin is leading to a reduction of the recruitment of CCR5-expressing cells in the murine TNBS- and DSS-induced colitis model.

## Materials and methods

### Animals

Our periostin-deficient mice (ko) that were described previously (11, 25) and wild-type littermates were initially used for this study in the BRF of the University of Southampton with the approval from the home office (PPL No. 70/8772). Furthermore, the project was continued in the facilities of Nazarbayev University with the Institutional Animal Care and Use Committee (IACUC) of the autonomous organization of education “Nazarbayev University” approval (15/30112020). The wild-type C57BL/6 mice used for the experiments in which no comparison between periostin-deficient (ko) and wild-type mice took place were obtained from «Masgut Aikimbayev’s National Scientific Center for Especially Dangerous Infections» of the Ministry of Healthcare of the Republic of Kazakhstan.

### DSS and TNBS mouse models

For TNBS colitis, 4–6-week-old mixed gender wild-type and periostin-deficient mice were anesthetized by intraperitoneal (i.p.) injection with ketamine/xylazine solution (100 mg/ml of ketamine and 20 mg/ml of xylazine in saline administered at 100 μl/10 g of body weight) and then treated with TNBS (0.5 mg per mouse) which was dissolved in 50% ethanol and administered intrarectally using a 3.5-French catheter equipped with a 1-ml syringe. To ensure uniform distribution of TNBS throughout the colon and cecum, mice were held in a vertical position for 60 s after instillation and then placed on a heated pad with bottoms up for up to 30 min. TNBS (picrylsulfonic acid solution) was purchased from Sigma-Aldrich (Cat. No. 92822). For DSS, the inflammation was induced by the oral administration of 5% DSS (MP Bio, colitis grade DSS, MFCD00081551) in drinking water, using 4–6-week-old mixed gender (predominantly female) of the corresponding genotypes for 6 days, and mice were sacrificed on the seventh day. Mice were randomly grouped and monitored daily: control (wt mice), periostin-deficient mice (ko), DSS-treated, and DSS in combination with orally delivered and intraperitoneally injected MVC (25 mg/kg/day) and intraperitoneally injected CCL5 5p12 5m (4.18 mg/kg/day).

### Immunohistochemistry and statistical analysis

For immunohistochemistry, the small intestine (DSS) and/or colon (DSS, TNBS) was embedded in OCT compound and sectioned at 10 μm thickness in CryoStar NX70 (Thermo Fisher Scientific, 2019). Sections were fixed in 4% PFA in PBS, washed three times with PBS, rinsed in PBS containing 0.1% Triton X-100, during 60 min blocked with 50% FBS, and incubated overnight with primary antibodies diluted 1:300 [CKR-5 antibody (D-6) CCR5-specific sc-17833, Santa Cruz; anti-periostin (ab14041), Abcam; recombinant anti-ROR gamma antibody (ab207082), Abcam] at 4°C. The sections were cleaned three times with PBS and then incubated with the 1:500 diluted appropriate secondary antibodies conjugated to Alexa 466 (Molecular Probes) or Cy3 (Jackson Laboratories; Chemicon) for 2 h and then washed and counterstained with 4′,6′-diamidino-2-phenylindole (DAPI; 0.001 mg/ml of PBS). The EVOS cell imaging system M5000 (Thermo Fisher Scientific) was used to examine and take pictures of the sections.

The immunohistochemical results were analyzed, and CCR5- or RORγT-expressing cells were counted in the different areas (from at least three or, in most cases, more independent animals) representing five and seven intestinal villi. The numbers of CCR5/RORγT-expressing cells were normalized to 100% in the wild type (+/+; wt) and compared to the numbers present in experimental cohorts. Data are shown as the average number of cells per area with standard error of the mean. Two-tailed, unpaired Student’s *t*-tests were calculated to compare each group. *P*-values below 0.05 were considered significant and flagged as *, while values below 0.01 were flagged with ** and values below 0.001 with ***.

### Histology

For histological analysis, extracted tissues were fixed in 4% PFA overnight, dehydrated by putting them into increasing amounts of ethanol, and then transferred to xylol. Afterward, the tissues were infiltrated with paraffin ON and afterward transferred to fresh paraffin for 1 h before embedding them in paraffin. The embedded tissue was cut into 5–8 μm sections and stained with hematoxylin/eosin.

### Drug supply and formulation

MVC (Pfizer) tablets (150 mg) were dissolved in sterile water and used as described (22). The total chemical synthesis of CCL5 5p12 5m was performed by Bachem, Switzerland (Lot no. 3013154); 2.5 mg of the preparation was resuspended in 20 mM of phosphate buffer pH 7.2, diluted, and used as described (22).

## Results

### Establishing a relation between periostin and CCR5 signaling

**Figure 1:**
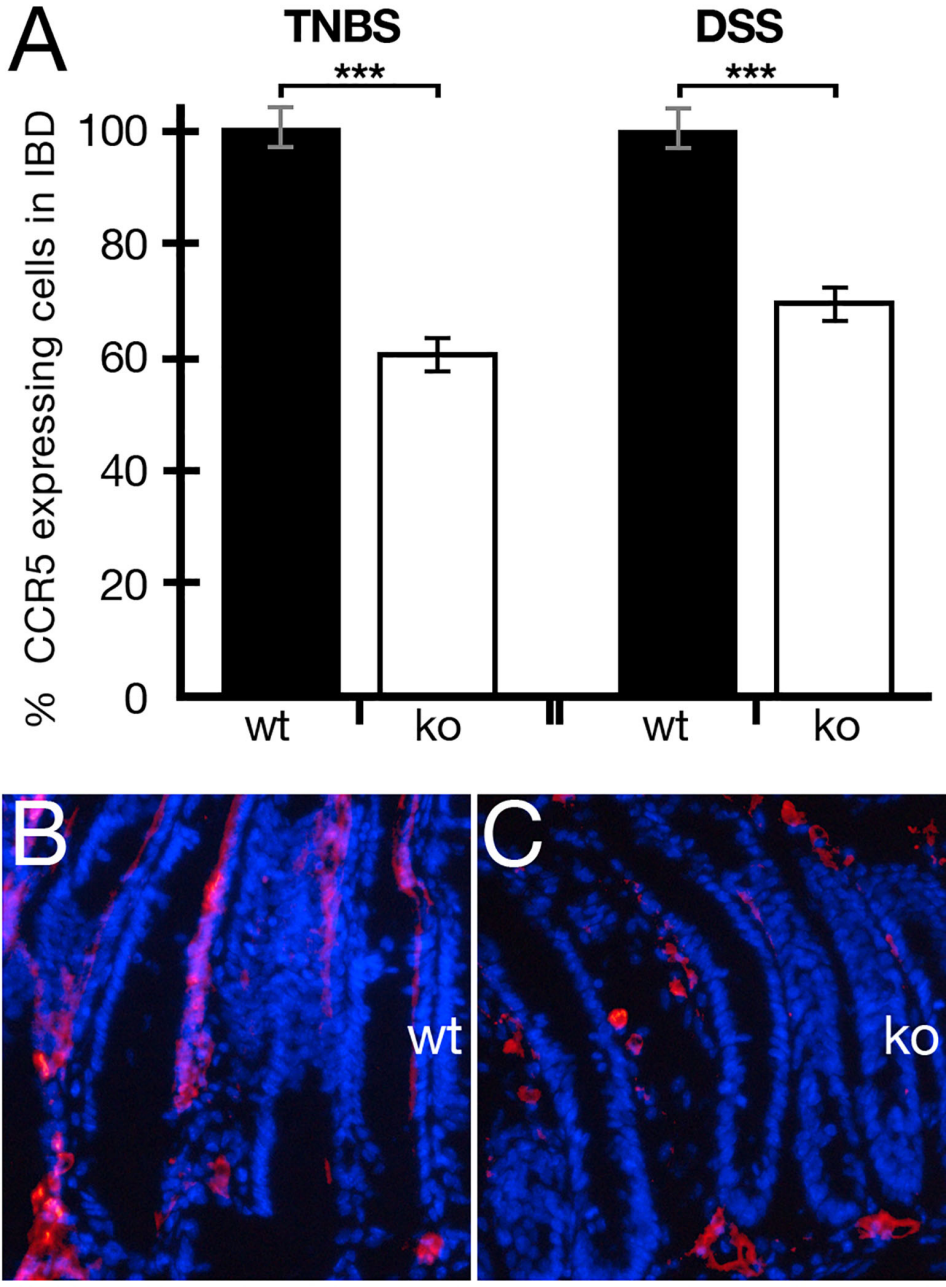
The absence of periostin reduces the number of CCR5-expressing cells in the colon/intestine of 2,4,6-trinitrobenzene sulfonic acid (TNBS)/dextran sodium sulfate (DSS)-induced colitis in mice. **(A)** The percentage of CCR5-expressing cells was normalized to wild-type mice (black histogram) and compared to periostin-deficient mice (white histogram). **(B, C)** Representative colon sections of the wild-type **(B)** and periostin-deficient **(C)** TNBS models. Immunofluorescence staining using the CDKR-5 antibody (red) and DAPI (blue). Please note the more regular appearance of villi in **(C)** supporting the clinical inflammatory bowel disease (IBD) scoring finding that periostin-deficient animals are more protected from chemically induced IBD. Please also note that CCR5-specific immunofluorescence captures of the small intestine in the DSS model are presented in **Figures 3C** (wt) and **(D)** (ko). At least three animals and between five and seven areas in at least three sections were used for determining the numbers for the graph in **(A)**. ***P < 0.001.

Our periostin-deficient strain (ko) (11, 25) as previously reported does not have any clinical or histological abnormalities except for incisor eruption. In their study from 2016, Koh et al. established periostin as an important player in the development and progression of chemically induced colitis using various models including periostin-deficient animals, recombinant periostin, and inhibitory antibodies directed toward periostin (17). We were able to confirm their previous finding that periostin is a driver of this disease and the absence of periostin is ameliorating the situation in chemically induced colitis in mice. We were interested in establishing a potential interaction between the periostin and the CCL5:CCR5 systems and monitored the expression of CCR5-expressing cells after IBD induction in wild-type and periostin-deficient animals. As can be seen in **Figure 1A**, the levels of CCR5-expressing cells in periostin-deficient mice were reduced significantly using the TNBS (40%) and the DSS (30%) model, compared to wild-type mice. Immunofluorescence analysis of colon samples (**Figures 1B, C** **)** shows a reduced number of CCR5-expressing cells in sections derived from TNBS-treated periostin-deficient mice in comparison to the wild type. Additionally, overlayed DAPI staining shows that the villi structure is more disorganized in the wild type (**Figure 1B**), highlighting the fact that mutant animals are showing less severe symptoms using chemically induced colitis models, as also previously reported (17). Reductions of CCR5-expressing cells in the small intestine in DSS-treated animals were analyzed and described in more detail in **Figure 3** also showing representative immunofluorescence sections (**Figures 3C, D** for wt and ko, respectively).

### Influence of periostin on immune cell recruitment to the intestine

**Figure 2:**
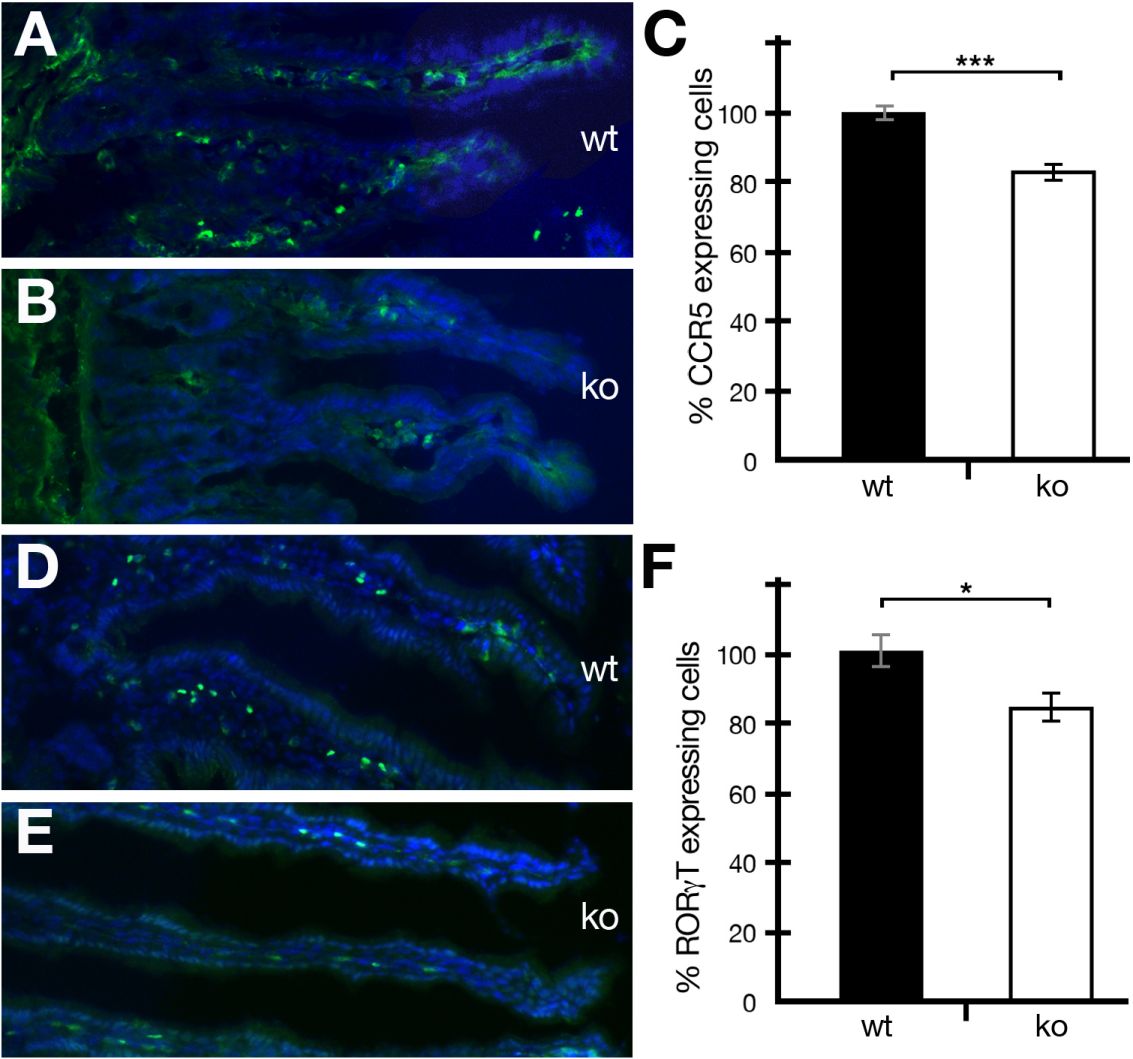
The absence of periostin reduces the number of CCR5- and RORγT-expressing cells in the intestine of healthy mice. **(A, B)** Representative captures of wild-type (wt) **(A)** and periostin-deficient (ko) **(B)** small intestine sections showing immunofluorescence staining using the CCR5 antibody (green) and DAPI (blue). **(C)** The number of CCR5-expressing cells was normalized to 100% for wild-type mice (black histogram) and compared to the numbers in periostin-deficient mice (white histogram). **(D, E)** Representative captures of wild-type (wt) **(D)** and periostin-deficient (ko) **(E)** small intestine sections showing immunofluorescence staining using the RORγT antibody (green) and DAPI (blue). **(F)** The number of RORγT-expressing cells was normalized to 100% for wild type (black histogram) and compared to the numbers in periostin-deficient mice (white histogram). Please note that the differences are highly significant (*P* < 0.001) in **(C)** and significant (*P* < 0.05) in **(F)**. At least three animals and between five and seven areas in at least three sections were used for determining the numbers for the graphs in **(C, F)**. *P < 0.05 and ***P < 0.001.

Periostin has been shown to be involved in a variety of different inflammatory diseases (12). However, no study on the effect of periostin on immune cells in a healthy uninflamed gut has been reported so far. The gut is a unique organ, containing a variety of different commensal microorganisms and other foreign materials. In order for gut homeostasis to be maintained, the right number and types of immune cells have to be present in the lamina propria of the intestine. We analyzed the occurrence of CCR5- and RORγT-positive cells in the intestine and found that both cell types were reduced in numbers in periostin-deficient mutant mice compared to wild-type controls (**Figures 2A, B, D, E** **)**. The reduction was around 16.8% in the case of CCR5^+^ (**Figures 2C**) and around 15.8% in the case of RORγT^+^ (**Figures 2F**) RORγT^+^ cells were readily detectable in healthy non-IBD-induced animals (**Figures 2D, E**).

### Influence of the absence of periostin and MVC on IBD

The application of MVC, a potent antagonist of CCR5, was able to ameliorate the disease phenotype in chemically induced colitis. The reduction in intestinal inflammation was at least partially achieved by selectively reducing the recruitment of CCR5-bearing leukocytes (22). In their study, a dosage of 25 mg/kg/day was sufficient to show beneficial effects, and thus, we decided to use the same therapeutic dose of MVC.

**Figure 3:**
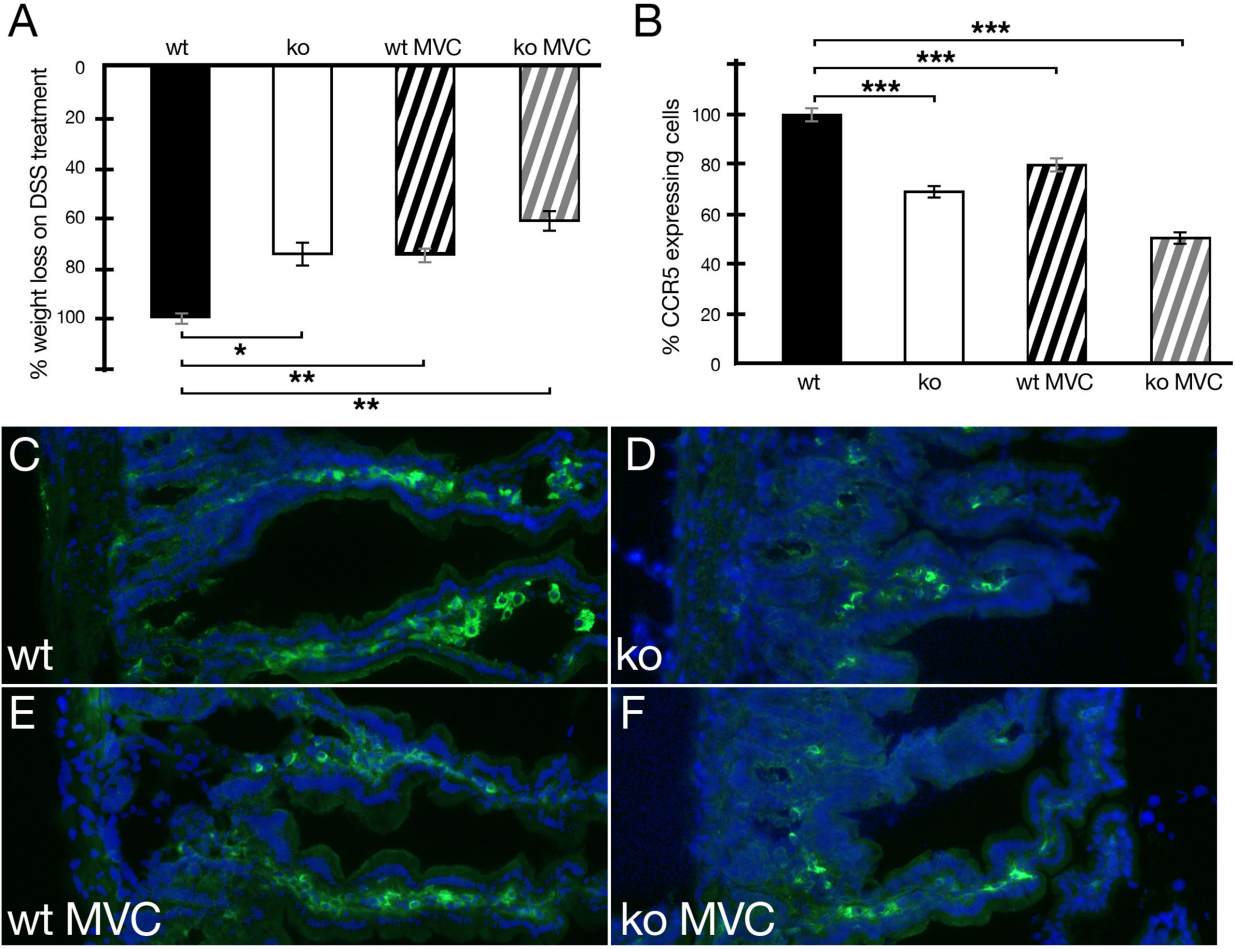
Weight loss of animals under DSS treatment with and without maraviroc (MVC) in drinking water in wild-type (wt) and periostin-deficient (ko) animals. **(A)** The weight loss observed in wt animals under DSS treatment was set to −100% (wt, black histogram). The weight loss as one read out of IBD severity for the wt cohort was −12.35% on average. The ko cohort (ko, white histogram) lost 9.37% on average and 9.39% on average for wt+MVC (black striped histogram) reducing the weight loss in both cohorts to 76%. The ko+MVC cohort (gray striped histogram) lost 7.79% on average leading to an overall weight reduction to 63% compared to wt animals under DSS treatment only. **(B)** Confirming our previous results, the absence of periostin results in the reduction of CCR5-expressing cells in the inflamed small intestine (−30%) (white histogram). The addition of MVC in drinking water has a similar effect (−20%) (black striped histogram). Interestingly, MVC in drinking water in periostin-deficient animals (gray striped histogram) has the strongest effect and suggests that the effects are additive (−45%). All differences in **(B)** are highly significant (*P* < 0.005). **(C–F)** Representative capture of small intestine sections from wt **(C)**, ko **(D)**, wt with MVC treatment **(E)**, and ko with MVC treatment **(F)** animals under DSS showing an immunofluorescence staining using a CCR5 antibody (green) and DAPI (blue). Immunofluorescence analysis shows the degree of reduction of the number of CCR5-expressing cells in sections derived from the inflamed small intestine in MVC-treated wt animals **(E)**, periostin-deficient animals **(D)**, and periostin-deficient MVC-treated animals **(F)** compared to sections derived from wt animals **(C)** under DSS treatment. At least three animals and between five and seven areas in at least three sections were used for determining the numbers for the graphs in **(A, B)**. *P < 0.05, **P < 0.01. and ***P < 0.001.

Next, disease progression in wild-type and periostin-deficient animals in DSS-induced IBD was tested in the presence or absence of MVC in drinking water. During daily monitoring, stool consistency, posture, provoked behavior, and evaluation of fur and overall appearance were generally slightly better in periostin knockout and MVC-treated experimental animals. This less severe phenotype was most clearly reflected in the less pronounced weight loss (**Figure 3A**). Both cohorts, periostin-deficient (ko) and wt MVC-treated mice, showed reduced weight loss (24%) compared to wild-type DSS-treated mice in the absence of MVC in line with previous reports (17, 22). Interestingly, the periostin-deficient mice (ko) treated with MVC showed the most beneficial effect with 37% reduced weight loss compared to wt in the absence of MVC. When analyzing the number of CCR5-expressing cells in sections of the small intestine in MVC-treated mice, we confirmed that periostin-deficient (ko) animals had lower numbers (30% lower) compared to the wild-type animals (**Figure 3B**). The addition of MVC in drinking water similarly led to a significant reduction in the number of CCR5-expressing cells in the inflamed small intestine (20% lower), compared to wt in the absence of MVC (**Figure 3B**). The difference in the level of CCR5^+^ cells was, however, less pronounced than in the cohort with the absence of periostin although the weight loss reduction was almost identical in both cohorts. Periostin-deficient mice treated with MVC showed the strongest reduction of CCR5-expressing cells (45% lower) when compared to untreated wt animals (**Figure 3B**). Representative immunofluorescence images of small intestine samples used to generate the data shown in **Figure 3B** are shown in **Figures 3C–F**. The strong reduction of CCR5-expressing cells observed in sections derived from MVC-treated periostin-deficient (ko) mice (**Figure 3F**) and untreated periostin-deficient mice (ko) (**Figure 3D**) in comparison to the untreated wild type (**Figure 3C**) can be easily observed, while the reduction in MVC-treated wt animals (**Figure 3E**) is less prominent.

### Influence of MVC and CCL5 5p12 5m on DSS-induced IBD in mice

**Figure 4:**
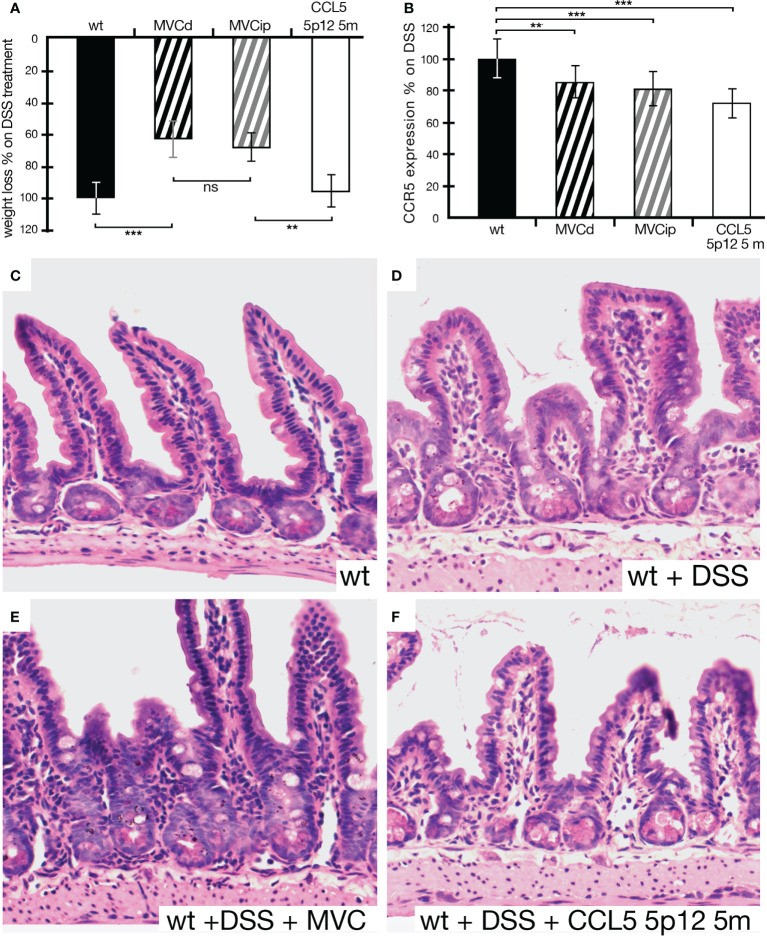
Weight loss of animals under DSS treatment being given MVC in drinking water, MVC i.p. or the CCR5 antagonist CCL5 5p12 5m. **(A)** As in **Figure 3**, the weight loss of wt animals under DSS treatment was set to −100% (black histogram, control). Upon treatment with MVC either *via* drinking water (black striped histogram) or i.p. injection (gray striped histogram), the weight loss dropped to 66% (MVC in drinking water) and 81% (MVC i.p.), respectively, indicating that both methods of application were able to decrease weight loss in DSS-induced colitis compared to control. While the difference in MVC treatment (in drinking water as well as i.p. injection) was highly significant, i.p. treatment with CCR5 antagonist (white histogram) did not result in a reduction of weight loss as one of the hallmarks for colitis severity. **(B)** The number of cells positive for CCR5 in DSS-treated wt mice was set as 100% (black histogram, control). In DSS-treated wt animals that were additionally given MVC in drinking water (black striped histogram) or i.p. injected (gray striped histogram), the weight loss dropped to 85% (MVC in drinking water) and 81% (MVC i.p.), respectively, when compared to that of the control. Treatment with the CCR5 antagonist CCL5 5p12 5m had the strongest effect decreasing the value to 73% of the positive cells found in the control. **(C–F)** Representative captures of small intestine sections from wt **(C)**, DSS-treated wt **(D)**, wt treated with DSS + MVC **(E)**, and wt treated with MVC + CCL5 5p12 5m **(F)**. At least three animals and between five and seven areas in at least three sections were used for determining the numbers for the graphs in **(B)**. **P < 0.01. and ***P < 0.001.

In order to test the protein-based CCR5 antagonist, CCL5 5p12 5m, we opted for the i.p. injection, previously used in the IBD setting (17). First, we compared MVC in drinking water to the i.p. injected MVC. As seen in **Figure 4A**, the i.p. injected MVC could ameliorate the effects of DSS-induced IBD compared to MVC in drinking water. However, CCL5 5p12 5m did not show any effect on weight loss, compared to animals that were given DSS in drinking water without any additional treatment (**Figure 4A**). Also, the other parameters observed during daily monitoring (stool consistency, posture, provoked behavior, and evaluation of fur and general appearance) did not reveal any improvement compared to the wild-type DSS control. Surprisingly, however, the number of CCR5-positive cells in these animals was even more reduced than in animals treated with MVC (in drinking water or i.p. injected) (**Figure 4B**). Macroscopically, the intestines isolated from MVC-treated mice (in drinking water or i.p. injected) had a better appearance compared to the untreated and the CCL5 5p12 5m-injected mice (data not shown). When analyzing the histology, the beneficial effect of MVC became apparent, and also the histology of the CCL5 5p12 5m-injected animals seemed slightly improved (**Figures 4C–F**).

This overall improved appearance can also be seen on the low power magnification of histological sections from all experimental groups seen in supplementary figure 1 (**Supplementary Figures 1A–D**).

## Discussion

It has been previously shown that homozygous mutant mice for periostin have less severe symptoms in a murine IBD model using DSS compared to wild-type mice (17). We could verify this in our study showing that the absence of periostin ameliorates inflammation in the TNBS- and DSS-induced IBD model. This is in good concordance with the fact that in both models the numbers of CCR5-positive cells in the intestine of periostin homozygous mutant mice were reduced compared to those of the treated wild-type littermates. A variety of different immune cells, among them T cells (T helper cells, cytotoxic T cells, and regulatory T cells), macrophages, and dendritic cells, express CCR5 (26). Our results in periostin homozygous mutant animals show that, in DSS-induced IBD, CCR5-positive cells were reduced by 30% and, in TNBS-induced IBD, even by 40%, compared to wild-type mice. This indicates that, in the absence of periostin, fewer CCR5-positive immune cells invade the intestinal tissue, possibly contributing to the reduced inflammation.

We next determined the number of CCR5-positive cells in untreated healthy animals. We could show that already in the unchallenged animals CCR5-positive cells were decreased by 16.8% in the intestinal tissue of periostin homozygous mutant mice compared to wild type. Histologically, no change in intestinal tissue could be observed (data not shown). Our mice were kept in individually ventilated cages (IVCs) and fed with sterile standard food. Therefore, although not germ-free, the microbial challenges in the intestine should be very low. Periostin-deficient animals do not seem more prone to dysbiosis or bacterial invasion of the gut, as also supported by the reduced number of immune cells in the unchallenged gut tissue. We are planning to challenge our mice in the future with different microorganisms and fungi in order to analyze whether periostin homozygous mutant mice will react differently compared to wild type in relation to inflammation and also tissue healing following the challenge (23).

Also, RORγT-positive cells were reduced by 15.8% when comparing periostin homozygous mutant to wild-type mice. RORγT is expressed mainly in Th17 cells, but also in Treg cells. As Th17 cells can be proinflammatory in nature, the equilibrium between effector Th17 and Treg cells is crucial for balancing intestinal homeostasis and inflammation (27). We also plan to analyze the differences regarding CCR5^+^ and RORγT^+^ cells more closely to understand whether specific subsets are affected or whether all subsets are affected equally and only the overall number of T cells in the intestinal tissue is reduced.

MVC, a drug used for the treatment of HIV, is a well-established CCR5 antagonist. It binds to CCR5 locking the receptor in an inactive conformation. It has been shown that MVC has a positive effect on murine IBD models by selectively reducing the recruitment of CCR5-bearing cells, leading to a reduction in inflammation (22). In this study, we compared the effect of MVC in DSS-treated wild-type and periostin homozygous mice. In concordance with previous reports, MVC ameliorated the symptoms of DSS treatment in wild-type mice, decreasing weight loss, affecting the clinical colitis scoring, and reducing the numbers of CCR5-positive cells in the inflamed intestine. We cannot formally rule out that also reduced expression of the analyzed protein, CCR5, in individual cells could result in picking up fewer signals by immunofluorescence analysis; however, as our data are in line with reductions published by Mencarelli et al. (22), we interpret our findings as a reduction in the number of cells expressing it. In periostin-deficient mice, this reduction was even more pronounced, indicating that MVC and the absence of periostin may have additive effects on the recruitment of CCR5-positive cells to the intestine.

A CCL5-based CCR5 antagonist, CCL5 5p12 5m, has been developed that presented an *in-vitro* 1,000-fold increase in inhibiting HIV-1 cellular infection as compared to MVC (24). We used this CCR5 antagonist to compare its effect in a murine IBD model and could show that the animals tolerated its i.p. injection. When DSS-treated mice were given CCL5 5p12 5m, compared to mice that were given MVC, even less CCR5-positive cells were present in the intestinal tissue, indicating that CCL5 5p12 5m is more effective in reducing CCR5-positive cell invasion of the intestine than MVC. Interestingly, this result was not reflected by the clinical colitis scoring, as animals did not show any significant improvement except for a slightly improved histology. Moreover, animals treated with CCL5 5p12 5m lost the same amount of weight as DSS-treated animals without any extra treatment. This indicates that other immune cell populations might be affected and the loss of a certain proportion of CCR5-expressing cells cannot solely account for the observed phenotype. However, it was claimed that MVC would not influence CCR2 signaling, a receptor reported to be susceptible to previously described CCR5 antagonists (28). It is known that under homeostatic conditions, the accumulation of monocyte-derived macrophages in the colon depends on CCR2 (29). In a recent paper using the murine colitis model, it was shown that the absence of CCR2 has differential effects in different contexts (e.g., using IL10R−deficient mice), demonstrating the complexity of the system (30).

The absence of periostin as well as the treatment with MVC results in a reduced weight loss and better clinical colitis scoring, while CCL5 5p12 5m does not. One possible explanation could be that CCL5 5p12 5m has more profound effects on the animal’s well-being and immune system. In order to investigate the potential effects of CCL5 5p12 5m, healthy animals should be thoroughly analyzed in the future. The higher anti-HIV-1 potency of CCL5 5p2 5m, as compared to MVC (24), is most likely a reflection of the extensive occupancy of the CCR5 active site cavity and thus higher specificity (31), a feature carefully demonstrated for a slightly less potent analog, 5P7-CCL5 (32).

In summary, we were able to show that periostin is contributing to the recruitment of CCR5-positive cells to the intestine in healthy as well as murine colitis models. The absence of periostin as well as MVC treatment helps to ameliorate the symptoms of chemically induced colitis. The positive effect of periostin deficiency in the IBD context can possibly at least partially be explained by the modulation of the CCL5:CCR5 axis. Both signaling pathways hold therapeutic potential and should be further exploited in future studies.

## Data availability statement

The raw data supporting the conclusions of this article will be made available by the authors, without undue reservation.

## Ethics statement

The animal study was reviewed and approved by Home office (PPL No: 70/8772) for the parts carried out in the BRF in Southampton, UK. Institutional Animal Care and Use Committee (IACUC) of the autonomous organization of education “Nazarbayev University” approval (15/30112020) was obtained for the parts carried out in the Nazarbayev University in Astana, Kazakhstan.

## Author contributions

SM, ES-R, LV, and DR contributed to the experimental and study design. SM performed the vast majority of experiments. AnB, AK, AiB, AA, and ES-R performed some of the experiments. SM, ES-R, and DR analyzed the data and prepared the manuscript. LV contributed to interpreting the results and critically edited the manuscript. All authors contributed to the article and approved the submitted version.

## Funding

This work was supported by grants from Nazarbayev University. In detail, these are Faculty Development Grants (FDCRGP) to DR (090118FD5310, 021220FD2751), to ESR (110119FD4510) and to LV (021220FD2551). Additionally, this work was supported by the Ministry of Health of the Republic of Kazakhstan under the program-targeted funding of the Ageing and Healthy Lifespan research program (IRN: 51760/ПЦФ-МЗ РК-19).

## Acknowledgments

We are grateful to Aida Kabibulatova, Zhuldyz Kairova, Zhanar Mustapova, Aisulu Nurmat, and Gulnaz Omarova for their great technical and administrative support.

## Conflict of interest

The authors declare that the research was conducted in the absence of any commercial or financial relationships that could be construed as a potential conflict of interest.

## Publisher’s note

All claims expressed in this article are solely those of the authors and do not necessarily represent those of their affiliated organizations, or those of the publisher, the editors and the reviewers. Any product that may be evaluated in this article, or claim that may be made by its manufacturer, is not guaranteed or endorsed by the publisher.

## Supplementary material

Supplementary Figure 1 Histology of small intestine following DSS induced colitis. The figure shows sections from small intestine at a low magnification from untreated wt **(A)**, DSS-treated wt **(B)**, wt treated with DSS+MVC **(C)** and wt treated with DSS+ CCL5 5p12 5m.
