# Supplementary figures and images for: Role of periostin in inflammatory bowel disease development and synergistic effects mediated by the CCL5–CCR5 axis
Source: Front Immunol. 2022 Oct 20;13:956691. doi: 10.3389/fimmu.2022.956691 (PMC9632729; doi:10.3389/fimmu.2022.956691)

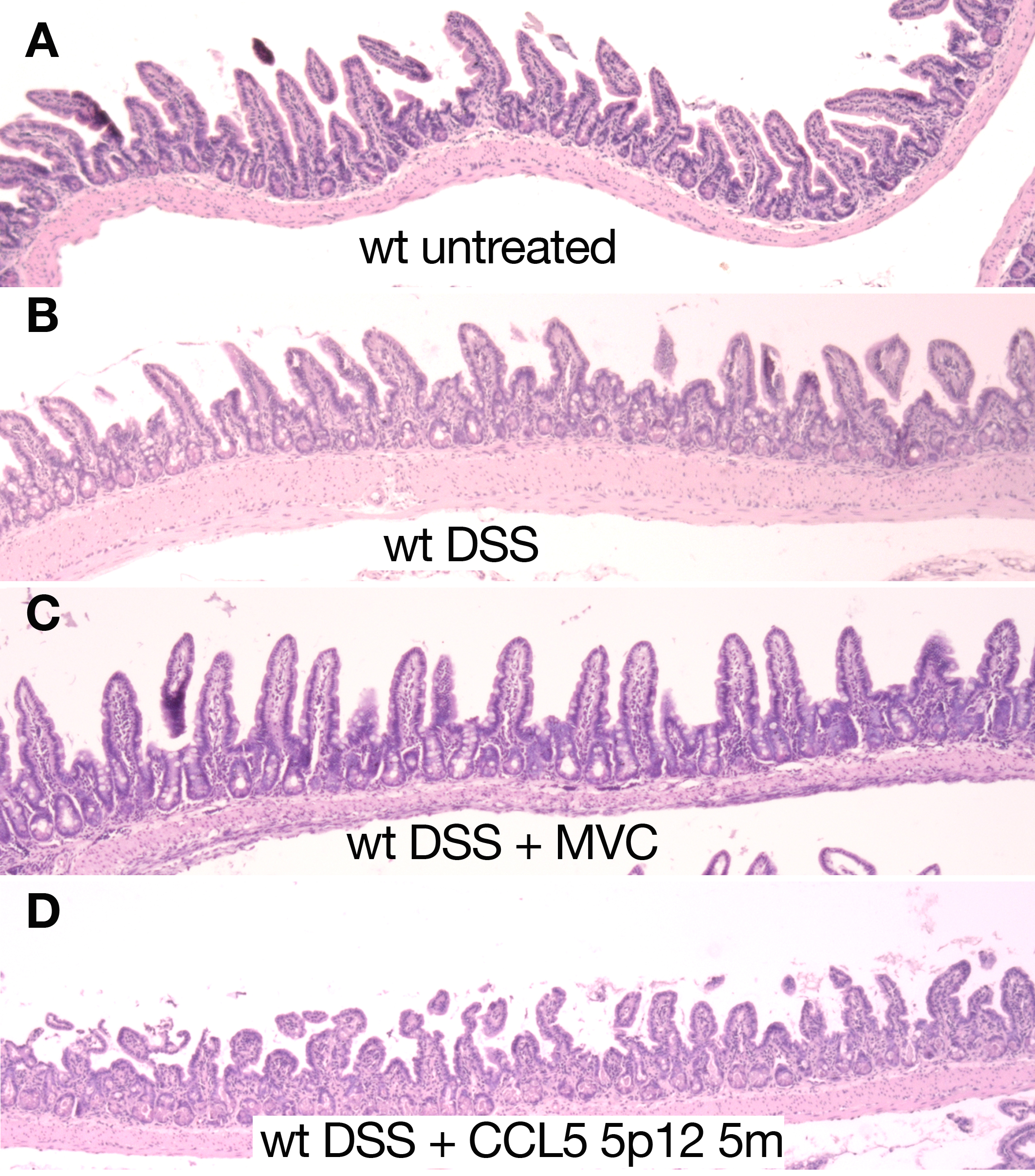

Supplement: Supplementary Figure 1 — Histology of small intestine following DSS induced colitis. The figure shows sections from small intestine at a low magnification from untreated wt (A), DSS-treated wt (B), wt treated with DSS+MVC (C) and wt treated with DSS+ CCL5 5p12 5m. [file Image_1.jpg]
